# Inaugural Address

**Published:** 1905-11

**Authors:** Willoughby D. Miller


### INAUGURAL ADDRESS.[1]

BY WILLOUGHBY D. MILLER, A.M., D.D.S., M.D., SC.D.[2]

THE provost of your university has done me the honor of inviting me to address a few remarks to you at the opening exercises of the new term of work and study which now lies before you. As I have the distinction of being an alumnus of the Dental Department, I felt that it might appear ungrateful for me to decline the invitation, and so I am here before you, notwithstanding many misgivings as to my fitness for the function which I have undertaken.

These initial exercises in which you are annually invited to participate appear to me to be of very vital importance. As you are all aware, every inert body set in motion has a tendency to go on moving forever in a straight line and in the direction originally imparted to it. And, in like manner, every receptive mind, such as that of the young student, has a decided inclination to follow along certain lines of thought or activity which have been imparted to it by those with whom it has come into contact. Hence

---

[1] Delivered at the opening exercises, October 2, 1905, of the twenty-eighth annual session of the Department of Dentistry, University of Pennsylvania.

[2] Professor University of Berlin, Germany.

we see how important it is that the young man, in setting out upon a career of whatever nature, receive his first impulses in the right direction, and how great a responsibility rests upon the one whose duty it is to impart these impulses.

Preliminary instruction or advice as to the general lines which a student should follow in acquiring his education, whether scientific, political, or ethical, might perhaps be better dispensed with here at the University of Pennsylvania than at many other institutions.

In foreign, especially German, universities, the student enjoys absolute freedom. He may attend the lectures or not, at his own discretion, and is obliged to pass no examination until he comes up for his degree. As a result, he may be, and sometimes is, matriculated at the university for months and even years without coming into contact with the teachers under whom he is supposed to be studying. This condition of affairs is exemplified by the anecdote of the man who, being asked the way to the university by a student, replied, "I don't know. I am a student myself." Here, the student is not only required to economize his time and to acquire habits of industry from the very beginning, but he is brought into closer relation with his teachers, and the opportunity of seeking and receiving advice from time to time is always present, and forms a safeguard of inestimable value if the student will but avail himself of it.

You, gentlemen, are particularly fortunate in having for your teachers and advisers a corps of men who are esteemed the world over as dentists, as scientists, and as men, and I have no better advice for you than that you put yourselves implicitly in their hands and follow their directions and their examples in every particular.

I have naturally no means of knowing what the motives were which determined each of you to take up dentistry as his life work. But of whatever nature they may have been, one thing is certain, that in acting upon them you have taken the most important step of your lives, a step, in fact, which fixes at once the whole configuration of your future lives, your occupation, your success, and to a great extent your happiness, and every one should be continuously awake to the fact that it is a very serious matter to take such a step, and, having taken it, he should bend all his energies toward successfully and honorably carrying it out.

A quarter of a century ago one of Germany's most famous surgeons, Professor von Mussbaum, characterized dentistry as a great and beautiful science. If this was true twenty-five years ago, it is doubly so to-day, for we may say, without fear of being accused of exaggeration, that, with the exception of bacteriology, no specialty of medicine has advanced more rapidly than, and very few as rapidly as, dentistry. Few accomplish as positive results, none repair injured or restore lost organs with the same degree of success, or with the same perfect imitation of nature, as is accomplished by the dentist. Moreover, in the same degree as a knowledge of the intimate and causal relations of the diseases of the teeth and mouth to the most diverse local and general disorders becomes more wide-spread, so, too, a more thorough appreciation of the importance of the science of dentistry is rapidly making its appearance everywhere, and especially in the ranks of the medical profession. It is, or soon will be, familiar to all of you that diseased conditions of the teeth are the cause of the most various local and general disorders of the human body. To say nothing of the inflammation of the dental pulp, of alveolar abscesses, periostitis, necrosis of the jaw, suppuration of the antrum and frontal sinuses, we know that neuroses, neuralgias, serious disorders of the digestive organs and of the air-passages and lungs, troubles of the ear, eye, and nose, as well as meningitis, septicæmia, chronic pyæmia, etc. very frequently have their origin in diseased teeth. More recently, moreover, Professor Bunge has shown that lactation is very markedly influenced by the condition of the teeth. The statistics of Röse and others reveal a striking percentage of recruits rejected because of bad teeth, and, what is of particular interest, various observers have found that the capacity of school children for giving attention to their lessons and for learning bears a direct relation to the condition of the teeth, and that good grades are indicative of good teeth. I need not dwell upon this point, for it is now universally known and acknowledged that the physical well-being of a nation, its progress and development, are in no small degree dependent upon its teeth; and we need not hesitate for a moment in placing the specialty which you have chosen, in respect to its capacity for relieving human suffering, for healing disease, and improving the general health and comfort, for intensifying capacity for work, physical or mental, on a perfect level with the other specialties of medicine.

A knowledge of all these facts has been for years, and still is, rapidly spreading among all classes of people, with the result that a demand for well-educated and skilful practitioners is rapidly increasing, so that the outlook for those who are now entering upon or already engaged in the study of dentistry is very encouraging. And with the opportunities which are afforded you here, it lies with great certainty in your power to attain success in your life work, with all the pleasures and comforts and power for doing good which accompany it. But you must not imagine for a moment that success comes of itself. It has been said that some men are born great, some achieve greatness, and some have greatness thrust upon them. But this does not apply to the dentist. The only way in which he can become great is by achieving his own greatness; and that he can do only by most diligent application and by taking advantage of every opportunity that is afforded him for perfecting himself in every branch of dental science and dental practice.

And you must not forget, gentlemen, that dentistry is a science so broad and comprehensive that to master it every moment of time at your disposal must be called into requisition, and anything short of that will not guarantee your success. It may entail your failure. For, while there is always room, and always will be room, for eminent workers in every profession and trade, there is but little place for the mediocre, and none at all for the inefficient or lazy.

It is said that the young are inclined to be optimistic, but this idea does not tally with the fact that our most pessimistic systems of philosophy of life, which are so well adapted to wreck the happiness of millions of otherwise contented beings, were brought out by men scarcely out of their twenties, while many of our most ardent exponents of a more hopeful and faithful view of life, have been men well advanced in years. I should say, therefore, that it is not optimism, but rather criminal thoughtlessness, when a student goes through his college days with no thought of the future, and doing only just the amount of work necessary to secure his degree. In fact, the student often makes the mistake of supposing that he is studying only for a degree, and that, the degree once obtained, everything else will come of itself. This is a great mistake, and the sooner it is remedied the better. The degree is simply an outward decoration, a guarantee that the holder has gone through with a certain amount of study and work; it adds nothing whatever to his capabilities.

Never forget that your chief object here is not to secure a degree, but to prepare yourselves for a great life work, and that your future happiness, success and position will depend upon the more or less perfect manner in which you carry out this object. Work always with this aim in view, and the degree will come of itself.

If you will permit me, I will give you in as few words as possible my conception of the ideal dentist of to-day.

In the first place, he must have a broad, liberal education to begin with, since it is folly to attempt to erect a beautiful superstructure upon a weak or rotten foundation. The dentist will be seriously handicapped in the pursuit of his professional studies if his liberal education and training have been deficient. A man, moreover, whose calling is at the same time scientific and humane, and brings him into contact with all classes of people, must be an educated man in order to uphold his own dignity and that of his profession. He must also be prepared to think and act intelligently in all social, political, and educational problems of the day.

The dentist must be thoroughly posted in all branches of general medicine which have a direct bearing upon dental science or practice. As a matter of fact, the question may be asked with perfect propriety, "Is there any reason why the dentist should not take exactly the same preparatory studies as the other specialists?" Certainly the relations of the teeth to the neighboring parts, and through the nerve-vascular and lymphatic systems to remoter parts, are quite as manifold and complex as those of the eye or ear; and why should the dentist be expected to know less of anatomy than the aurist or ophthalmologist? The same question may also be asked in respect to physiology, hygiene, general pathology, bacteriology, etc. I personally would say, in answer to this question, that the science of medicine in all its branches is growing so rapidly, and has already become so extensive, that no one, even though he may have five, ten, or even twenty years for study, will ever be able to master all parts of it equally well, and that it will be expedient if not necessary in the future that every specialist restrict himself more or less to those branches of study which have a direct bearing upon his own specialty. There is no more reason for the aurist than for the dentist to devote months to acquiring a thorough knowledge of obstetrics or gynæcology, or even of the anatomy of the extremities, physiology of the spleen, etc. On one ground only we may be possibly justified in limiting the general medical studies of the dentist somewhat more than those of other specialists, and that is because he requires more time than they to master his own special studies and operations. We must, however, carefully avoid going too far in this direction, since, as already indicated, to use a geographical expression, dentistry is absolutely bounded on all sides by the science of medicine, and must stand on the same or at least on as firm a foundation as the other branches of medical science.

The dentist must naturally be particularly proficient in all special subjects relating to the science and practice of dentistry. He must also possess a high degree of manual dexterity, in view of the exceeding delicacy required for the carrying out of many operations which he is called upon to perform. The want of this dexterity has been the cause of the failure of many dental practitioners, and the student who is not endowed with it by nature should make every effort under the direction of his instructors to acquire it. He must not forget that dental operations are to many patients exceedingly painful, and he should take every precaution to reduce their discomforts to a minimum. The idea that the excavation of a tooth, to be perfectly done, must of necessity be painful always seemed to me to be a bit barbarous, and sometimes made use of as a mantle to cover lack of care or the use of improper instruments.

The dentist must be a most strict observer of the fundamental principle of modern surgery,—*i.e.*, of the rules of asepsis. In no department of medicine is a demand for asepsis—*i.e.*, cleanliness—greater than in the practice of dentistry, both on account of the danger of transmitting diseases and because the dentist is brought into more intimate contact with his patients than the general practitioner. The student of dentistry should, accordingly, from the very beginning, cultivate habits of the strictest cleanliness, not only in respect to his person, especially the hands, but also to his napkins, his instruments, materials, and everything which comes into contact, directly or indirectly, with the mouth of the patient. An experience of twenty-one years of teaching has taught me that one cannot insist too strongly or too often upon this point. He must studiously avoid getting into the habit of degrading his profession to the level of a trade, or of putting to himself the question, "How many dollars and cents are there in this mouth for me?" instead of "How can I best save these teeth and make them comfortable and useful to the patient?"

No dentist can conduct a practice on such a principle without dishonoring himself and his profession. Certainly it is right and honorable that he receive a fair remuneration for his work. It is right that he should accumulate enough of wealth during the long years of a responsible and trying practice to enable him to meet old age free from care. But to make everything else subordinate to the one thought of getting money, and possibly to sacrifice the best interests of those who intrust themselves to his care, for this aim, is to do violence to the ethical principles by which every member of the humane professions is inexcusably bound, and the dentist who conducts his practice on this plan is little better than a parasite.

I may also say in this connection that an institution which exists ostensibly for the purpose of preparing men for the practice of dentistry, but which sacrifices what is acknowledged to be the best interests of its students in favor of a greater balance in the cash account, deserves no better consideration, and the sooner it goes out of existence, the better for all concerned.

The dentist should not be over hasty to criticise the work of a colleague, as he cannot know the exact conditions under which it was done, nor can he by any means always trust the statements of the patient in regard to it. Above all things he should never, by a shrug of the shoulders or by silent acquiescence in erroneous statements of the patient, sanction an unjust accusation against a colleague. No dentist who has a proper feeling of self-respect would do a thing so mean, to say nothing of the fact that the patient usually sees through the object of it. On the contrary, it is the simple duty of every dentist to strongly and unequivocally defend the character of a brother dentist when he sees that it has been unjustly attacked.

There is still another matter to which I wish to call attention. The patient-material upon which the student operates in the dental college is recruited largely from the poorer classes,—servants, day-laborers, etc.,—and the student easily acquires the habit of thinking that it is not necessary for him to have the same respect of person, or to carry out the treatment with quite the same care or consideration, that he would observe in his private practice. This is a grave mistake, for habits acquired during your college practice are difficult to change later on, to say nothing of the fact that the poor man has the same sensibilities, and, as patient, is entitled to just the same respect as the rich man. You should, accordingly, in your college practice, observe in every particular the same degree of care as will be demanded of you in your private practice later on.

I have been in the habit of pointing out to my students a certain criterion by which they, in a way, are enabled to foretell their success or lack of success in their future practice.

If the patients are satisfied with the treatment which they have received, and if, on returning later on, they ask to be treated by the same student, and bring their friends with them for treatment, so that the student in a way accumulates a practice about him in the dental college, I do not hesitate to say that his future success is assured.

If, on the other hand, patients complain of his treatment and apply to the demonstrator to be put into the hands of some other student, then it is time for him to seriously inquire whether he is not in some vital respect at fault, and to give himself the greatest pains to remedy the fault; for if he is not able to give satisfaction in his college practice, he will do it much less later on.

Do not forget that you have duties to perform in the interest of your profession. A member of a family, a society, community, or profession cannot isolate himself from his surroundings, or honorably escape from the performance of his duties toward the organization of which he is a part. The dentist owes all he has to his profession, and if he does not seek to make some return, he is either very selfish or, at best, very thoughtless. It is his duty to support the societies which are the nerve-centres of the profession. He should do all in his power to advance dental science. He should extend his aid and encouragement to the less fortunate brothers, and help to elevate the standard of his profession by living a righteous and just life and by seeking the advancement of his profession as well as his own. Above all things, he should not make use of any influential position which he may have acquired in any dental organization for carrying out his own devices.

A young man beginning the practice of dentistry should be prepared to undertake the treatment of all diseases of the teeth and contiguous parts which present themselves; excepting, of course, surgical cases requiring a more special training than he has been able to acquire, which he should refer to some colleague who has made a specialty of such operations. It does not follow from this, however, that it may not be advantageous for him, as well as for his patient, to restrict his practice to some particular branch of dental surgery; in fact, the practice of dentistry has become so extensive and is growing so rapidly that the time will soon come—if not already here—when it will not be advisable for the dentist to attempt to do all operations which come within the scope of his profession. And if you find that you have a special aptitude for any particular work, whether it be for the preservative treatment of the teeth, for dental surgery, for prosthetic dentistry, for crown- and bridge-work, or porcelain, give your particular attention to such work, if circumstances permit, and eventually you may be able to restrict yourself to that alone, with the result that you will work with much more satisfaction and less strain to yourself, and serve your clients better.

Great opportunities are offered to the young man now entering upon the practice of dentistry. Our profession is as yet only in its infancy, and the work so far accomplished, great as it is, is small in comparison with that which is still to be done. At present the great aim of medical science is to prevent disease by restricting its courses, by assisting the body in the use of those powers of resistance with which it is naturally endowed, and by inciting such powers where they are wanting, and we, as dentists, following out the same line of thought, have our problem of immunity to caries, as observed in individual persons to deal with, and the practically still more important problem growing out of that of conferring immunity upon persons naturally disposed,—*i.e.*, the problem of the prevention of the decay of the teeth,—and the man who succeeds in solving this problem will confer a greater boon upon humanity than resulted from the discovery of the X-Ray, or of the diphtheria heil-serum.

Another great problem with which you will have to grapple is that of making the blessings of dentistry accessible to the great masses of the people. Dental operations as now carried out are disagreeable, if not absolutely painful, to the patient, as well as trying to the operator, and, in particular, they require an expenditure of time which appears to be out of proportion to the operation itself. We consume more time in filling a simple cavity in a tooth than the surgeon in amputating a leg or performing a laparotomy, and when we have to do with a pulpless and diseased molar, the amount of time spent may be five to ten times as great as is necessary for a difficult surgical operation upon which the life of the patient is depending. The result is that the care of the thirty-two permanent teeth becomes a very expensive matter, and many a father of a family pays more to the family dentist than to the family physician,—*i.e.*, more for the care of the teeth than for that of all the rest of the body together. We are also forced to admit that for the same reason the blessings of preservative dentistry are a luxury far beyond the reach of the great masses of the people. Taking it the world over, not one person in five hundred could afford the expense of having his dead molars properly cared for. In European countries the percentage will be about one to twenty-five or thirty. In America, where there is much more money in circulation among the middle and labor classes, it may possibly be as high as one to five. Nor does it appear to me that the development of dentistry in the last few years has been such as to make it more accessible to that class of people which in the daily struggle for existence suffers much more from the lack of sound teeth than the opulent classes who stand above the struggle and are better able to compensate for the lack of sound teeth by other means. At present dentistry is developing most rapidly in the artistic direction, and there is a great cry for artistic operations. Everything must be made of porcelain,—porcelain bridges, porcelain crowns, porcelain fillings,—and everything carried out with such a highly developed sense of the artistic that the artificial nature of the work shall be absolutely undetectable to the unskilled eye.

And we must admit that there is a great future for the dentist in this class of operations, and great credit is due to all who have done anything to perfect this particular line of work, and it is to be hoped that the time is not far distant when the gold crown on an incisor root will be a thing of the past. But, nevertheless, this class of work tends to render dental aid still less accessible to the poorer classes, and its particular culture cannot be regarded as an unmixed good unless at the same time an equal development takes place along other lines.

We need materials and methods which will render it possible to shorten and cheapen dental operations so as to bring them within the reach of every one. We need, accordingly, a filling-material of the nature of a cement which can be quickly and easily inserted and will preserve the teeth permanently. We need some way of shortening and simplifying our present long and complicated methods of treating root-canals, and the one who discovers and introduces such material and methods will confer a greater boon upon humanity than those who have contributed to the development of the art of employing porcelain in the preservative treatment of the teeth, for we must always bear in mind that it is the object and evident duty of our as of every other humane profession to do the greatest possible good to the greatest possible number of people.

Professors of a great and beautiful science with a constantly widening sphere of activity, with manifold great and complex problems, both scientific and practical, to be solved, a great future lies before you, and if you are willing, I doubt not that you will also show yourselves capable of meeting all the requirements that your profession justly exacts of you. Liberally educated, well posted in all collateral branches of medical science, master in the science and practice of dentistry, unerring in the performance of his duties, ethical and Christian to his patients, his colleagues, his profession, and his country,—this is my conception of what the dentist ought to be, and of what I trust each of you will be, never forgetting that, however great a thing it may be to be a good dentist, it is far greater to be a good man.

